# The new platforms of health care

**DOI:** 10.1038/s41746-021-00478-5

**Published:** 2021-07-15

**Authors:** E. Ray Dorsey

**Affiliations:** Center for Health + Technology and Department of Neurology, University of Rochester Medical Center, Rochester, NY USA

**Keywords:** Health services, Health policy

## Abstract

For the past century, health care measurement and delivery have been centered in hospitals and clinics. That is beginning to change as health measures and increasingly care delivery are migrating to homes and mobile devices. The COVID-19 pandemic has only accelerated this transition. While increasing access to care and improving convenience, this move toward platforms operated by for-profit firms raises concerns about privacy, equity, and duty that will have to be addressed. In addition, this change in measuring health and delivering health care will create opportunities for educators to expand the settings for training, researchers to conduct studies at enormous scale, payors to embrace lower-cost clinical settings, and patients to make their voices heard.

For the past century, the dominant locations for assessing health and delivering health care have been clinics and hospitals. That is about to change (Table 1).

**Table 1:** Comparison of health care platforms.

Characteristic	Clinic-based	Technology-based
Principal measurers of health	Clinicians	Devices
Health measurements	Primarily subjective	Primarily objective
Frequency of measurements	Episodic	Continuous
Location of visits	Artificial like the clinic	Real world including the home
Nature of visits	Primarily synchronous	Synchronous and asynchronous
Clinicians	Few	Many
Limiting factors	Social and geographical	Social and technological
Concerns	Access, quality, and cost	Privacy, equity, and duty

In *The Third Wave*, Steve Case, the co-founder of AOL, posited that the internet was poised to “transform major, real-world sectors” like health care^1^. The coronavirus disease 2019 (COVID-19) pandemic has accelerated that transformation through the rapid adoption of telemedicine and remote monitoring. The platforms underlying these new approaches to health, however, are from technology firms, not medical centers.

## Use of digital devices to measure health and deliver health care

Like the nervous system, these platforms enable both afferent (sensing) and efferent (acting) information flows. The afferent paths measure health through an increasing array of digital devices, including portable (smartphone), wearable (watches), and residential (virtual assistants). These devices can now detect abnormal heart rhythms, blood glucose levels, cognitive impairment, and seizures. They do so by capturing objective, frequent data in the real world in contrast to the often subjective, episodic data gleaned from a clinic visit. The results are new health insights and likely earlier and more frequent detection of disease. These platforms are expanding in scale and scope. For example, Alphabet recently completed its purchase of the wearable device company Fitbit, and Facebook has announced plans to build a smartwatch with a fitness focus that can capture health data.

The efferent paths are less developed but are beginning to emerge. The Apple Heart study enrolled 40,000 individuals who had a smartwatch to determine whether occult atrial fibrillation could be detected^2^. If a suspicious heart rhythm was found, the participant was connected to a remote physician from a telemedicine company. As the study illustrated, the big technology firms may not be involved in actual care delivery, but their tools will increasingly serve as the vehicles over which care is provided. All of these moves are part of a broader push by the largest technology firms in the U.S. and China (e.g., Tencent) to invest in health (Table 2). This activity is going beyond hardware and includes investments in clinician-facing software, care delivery, and even health insurance. As big as these companies are, the largest (Apple) has a market capitalization ($2.0 trillion) that is less than what the U.S. spends ($3.8 trillion) on health care per year.

**Table 2:** Select investments in health by the largest technology firms, 2019–2021.

Company	Market capitalization ($ billions as of May 12, 2021)	Recent activities
Apple Inc.	$2040	• Collaborated with Eli Lilly and Evidation Health in joint study to detect cognitive decline • Expanded capabilities of smartwatch to include measuring blood oxygen levels • Enabled U.S., U.K., and Canadian providers to share health data on its mobile Health Records application
Microsoft	$1800	• Partnered with Providence St. Joseph Health to build a “hospital of the future” in Seattle • Developed Microsoft Cloud for Healthcare, first industry-specific cloud solution • Acquires speech-recognition company Nuance for $20 billion
Amazon.com	$1590	• Created and expanded Amazon Care for its employees and other companies • Launched its own fitness tracker, Halo • Expanded home monitoring features of Alexa to include a “Care Hub” to facilitate aging in place
Alphabet Inc.	$1490	• Invested $100 million in Amwell, a large telehealth company • Saw two of its investments in health insurance start-ups, Oscar Health and Clover Health, become publicly traded companies with multi-billion dollar valuations • Finalized $2.1 billion acquisition of Fitbit
Facebook, Inc.	$860	• Launched new Preventive Health application • Acquired a technology company that can control a virtual hand • Plans to launch a wearable health tracker in 2022

The technology giants are not the only ones entering health as new companies are seeking to develop novel approaches to care. In 2020, venture capitalists and others invested $21.6 billion in digital health companies, several digital health companies had highly successful initial public offerings, and a merger between a telemedicine (Teladoc) and a disease management (Livongo) company resulted in a $36 billion firm. At the same time, telemedicine use soared. In just 1 month, telemedicine visits among Medicare beneficiaries increased 100-fold^3^. At the pandemic’s peak, telemedicine accounted for most patient visits at many medical centers. In the span of weeks, we saw the greatest transformation in health care delivery in the past 50 years.

These developments are part of a broader migration of care from hospitals and clinics to home and mobile devices^4^. Everything from acute stroke care (mobile stroke units^5^) to hospitalizations for pneumonia (hospital at home^6^) are moving toward the home. This transition mirrors what has already occurred in other industries, such as entertainment, banking, and retail. Underlying these transitions are mobile devices, which are amazingly young. The smartphone, first released in 2007, is still a teenager.

## Benefits and limitations of health care’s new platforms

The benefits to these platforms are potentially substantial. First, they can capture enormous volumes of data in a person’s natural environment. Rather than dosing insulin based on finger sticks obtained in doctor’s office, insulin can now be titrated based on continuous measurements directly from an individual. Even in the absence of closed loop systems, these large pools of data have value. They can give individuals more control over their health, foster a sense of self-efficacy, and identify previously occult features of a disease. Second, because smartphones, watches, and even home monitoring devices are increasingly ubiquitous, these tools can connect individuals to care. This care can come from a wide range of clinicians who are difficult to organize in traditional clinics but can reach patients via synchronous and asynchronous encounters. Most individuals receive too little care and face significant obstacles to receiving appropriate treatment. This is especially true in lower-income nations where clinics are sparse and smartphones are ubiquitous. Finally, such care can be centered around the needs of individuals rather than institutions. The most consistent finding from telemedicine studies is high patient satisfaction^7^.

While the benefits are great, so are the concerns around privacy, equity, and duty. The data captured by digital devices can inform countless aspects of behavior and health. These data could be valuable to marketers, employers, and governments. Just as advances in genetics required policy protections for individuals, so too do advances in technology. In 2008, the Genetic Information Nondiscrimination Act was hailed by the late Senator Edward Kennedy as the “first major new civil rights bill of the new century.”^8^ A similar act for data is needed to avoid discrimination and to recognize explicitly that individuals have the right to determine who can see their data and for what purpose^9^.

Current health care is plagued by inequities^10^. While technology platforms hold the potential to make care more accessible, the results to date have been mixed. Twenty percent of households in the U.S. lack broadband access, and a similar proportion do not have a smartphone. Broadband access should become a public good available to all, and these new platforms should seek to bridge rather than expand the current gaps in health^11^. To do so, some individuals will require assistance, either remotely or in person, to use these new technologies at home^12^. Alternatively, telemedicine can be provided in clinics or other locations (e.g., libraries) near patients’ homes. All will benefit from simpler tools, some of which could be provided directly to individuals, that almost anyone anywhere can use^13^.

Finally, the technology companies that own these platforms are accountable to shareholders, not to patients or the public. Shareholders may be, and likely are, less concerned with privacy and equity than individuals or the broader society. Professions have a duty to the individuals they serve. Non-profits are ultimately accountable to the public. Technology firms do not share these responsibilities. If their devices will be the new platforms for measuring health and delivering care, the interests of individuals must be protected.

## Opportunities and next steps

This migration will require action from all of us—educators, researchers, payors, and patients. As care moves toward the home and medical devices, medical training will have to prepare future physicians and other clinicians for settings beyond the hospital. Unfortunately, such training is almost absent. In a survey conducted 20 years ago, simple lectures on home care did not exist in half of internal medicine residency programs, and only a quarter of residents had a mandatory house call experience^14^. Training, exposure, and experience with telemedicine may be worse. Only one specialty (child and adolescent psychiatry) even mentions telehealth in its list of competency-based outcomes^15^. Educators need to prepare trainees for health care’s future, one that will bring care to patients rather than patients to care.

The first step to that preparation is to expand training outside the hospital. If care is migrating to homes and mobile devices, so should trainees in most clinical disciplines and at all levels. House calls, the gold standard of patient-centered care, should be experienced by all students and more than once (as is the requirement where I work). Similarly, telemedicine should be part of clinical training from the beginning. The scale and scope of these experiences can expand from common synchronous one-to-one encounters to one-to-many group visits to many clinicians connecting to a single patient for complex care management. Similarly, the complexity and demands of telemedicine can increase with training to include support of other clinicians (e.g., through remote intensive care units or asynchronous consultations). Reflective exercises that ask trainees to consider the relative benefits and limitations of these emerging care models from the perspectives of patients, clinicians, and payors could be illuminating.

Researchers must be far more ambitious in the scale and scope of their investigations. In 2015, Apple’s open-source ResearchKit demonstrated the ability of smartphones to enroll thousands of research participants in a single day^16^. Today, the National Institutes of Health’s All of Us Research Program aims to enroll at least one million Americans in an observational study that includes questionnaires, clinical assessments, collection of biological specimens, and use of digital health technology^17^. Verily’s Project Baseline study is embracing these platforms even more to recruit, enroll, and assess research participants with multiple digital devices, including a smartphone, a sleep sensor, and a smartwatch^18^. The combination of the in-clinic and at-home assessments in these studies will provide a much deeper phenotype of disease and health than is available in traditional, episodic clinic visits.

Future efforts can go farther. Why take until 2024 to enroll one million participants?^17^ Why limit such studies to just Americans? In general, people want to participate in research, to advance knowledge, and to help accelerate therapeutic development for diseases that affect them or their families^19^. Decentralized research studies can bring research studies to participants and utilize smartphones and other digital devices that over 40% of the world’s population now possess^20^. Social media and patient registries are powerful tools for recruiting potential research participants, the vast majority of whom are not seen at traditional research sites^21^.

Insurers, public and private, need to embrace care models that are centered in safer, more patient-centered, and less expensive settings than the hospital^6^. For example, Medicare’s expanded coverage of telemedicine by geography (e.g., outside of health professional shortage areas), setting (e.g., the home), and clinicians (e.g., speech therapists) enabled its rapid adoption. However, these changes, motivated by an infectious pandemic, are temporary. Whether Congress makes them permanent will be one of the great determinants of future health delivery in the U.S. and is far from guaranteed. Major medical centers and hospital chains benefitted from the ex ante where reimbursement was rich for in-person care and often absent for remote care. The rationale for facility fees for expensive, institution-centered care that is geographically and socially distant from underserved communities is unclear. Promoting parity in reimbursement regardless of a patient’s location will help advance new care models and make care more accessible.

Private insurers, previously concerned about excess utilization, may follow Medicare’s lead. Many, including insurers (e.g., Cigna) that have purchased telehealth companies (e.g., MDLive), are betting on such a future. Similarly, the need to expand home care coverage will be required to satisfy the preferences of the three quarters of Americans who want to live in their own homes as long as possible^22^. This desire is only likely to be heightened by the tragic toll that COVID-19 took on residents of nursing homes.

The fate of many of these changes will be ultimately dictated by individuals in their capacity as patients, research participants, and citizens. Individuals pay for health care coverage either through employment or taxes. Studies rely on the goodwill and trust of research participants to occur. Citizens and their representatives determine the financial incentives that educators and clinicians face. The past 2 years have highlighted many of the substantial shortcomings of institution-centered care and provided powerful glimpses of the alternatives that measure health and deliver health care outside of hospitals and clinics. The principal beneficiaries of these new models are not the providers of care and research but the recipients. These individuals will have to make their voices heard for progress to continue. Proponents of institution-based care certainly will.

## Conclusion

A century ago, advances in transportation (e.g., cars and roads) and technology (e.g., electrocardiograms, x-rays) drove care away from homes and toward hospitals and clinics. Today, advances in technology are reversing that trend. The digital devices that we carry, wear, and have in our homes are now measuring our health constantly and will soon be the means by which we receive care. The infrastructure is now in place (81% of Americans own a smartphone; 20% wear a fitness tracker)^23^, the capabilities of these devices are increasing, and the desire and need for care in the home are rising. The promise is that our measurement of health will improve and that care will be more accessible. However, for that to occur, we will need to establish new rules for the road and ensure that these platforms are available to all.
